# Greater Biomass Production Under Elevated CO_2_ Is Attributed to Physiological Optimality, Trade-Offs in Nutrient Allocation, and Oxidative Defense in Drought-Stressed Mulberry

**DOI:** 10.3390/antiox14040383

**Published:** 2025-03-25

**Authors:** Songmei Shi, Huakang Li, Xinju Wang, Ziran Wang, Junqiang Xu, Xinhua He, Zheng’an Yang

**Affiliations:** 1 Key Laboratory of Vegetable Biology in Yunnan, College of Landscape and Horticulture, Yunnan Agricultural University, Kunming 650201, China; shismei2022@ynau.edu.cn (S.S.); 2023210206@stu.ynau.edu.cn (H.L.); 2022210207@stu.ynau.edu.cn (X.W.); wangziran@cau.edu.cn (Z.W.); xujunqiang101@163.com (J.X.); 2 Centre of Excellence for Soil Biology, College of Resources and Environment, Southwest University, Chongqing 400716, China; 3 College of Forestry, Sichuan Agricultural University, Chengdu 611130, China; 4 School of Biological Sciences, University of Western Australia, Perth, WA 6009, Australia; 5 Department of Land, Air and Water Resources, University of California at Davis, Davis, CA 95616, USA

**Keywords:** antioxidant mechanisms, chlorophyll fluorescence, leaf photosynthesis, nutrient uptake, oxidative stress, plant biomass

## Abstract

Mulberry (*Morus alba* L.), a species of significant ecological and economic importance, is widely cultivated for sericulture, soil conservation, and environmental restoration. Despite its remarkable resilience to environmental stresses, the combined impact of elevated CO_2_ (eCO_2_) and drought stress on aboveground–root–soil interactions remains poorly understood, particularly in the context of global climate change. Here, we investigated the effects of eCO_2_ and drought on physiological leaf and root indicators, nutrient absorption and allocation, and soil properties in mulberry seedlings. Mulberry seedlings were grown in environmentally auto-controlled growth chambers under ambient CO_2_ (420/470 ppm, day/night) or eCO_2_ (710/760 ppm) and well-watered (75–85% soil relative water content, RWC), moderate-drought (55–65% RWC), or severe-drought (35–45% RWC) conditions. Results showed that both above- and below-ground plant biomass production were significantly promoted by eCO_2_, particularly by 36% and 15% under severe drought, respectively. This could be attributed to several factors. Firstly, eCO_2_ improved leaf photosynthesis by 25–37% and water use efficiency by 104–163% under drought stresses while reducing negative effects of drought on the effective quantum yield of PSII photochemistry and the photochemical quenching coefficient. Secondly, eCO_2_ significantly decreased proline accumulation while increasing soluble sugar contents, as well as peroxidase and superoxide dismutase activities, in both leaves and roots under drought stress. Lastly, eCO_2_ promoted soil sucrase, urease, and phosphatase activities, as well as plant nitrogen, phosphorus and potassium uptake while facilitating their allocation into roots under drought stress. These findings demonstrate that eCO_2_ enhanced the drought tolerance of mulberry plants through improvements in photosystem II efficiency, water use efficiency, antioxidative defense capacity, and nutrient uptake and allocation, providing critical insights for sustainable mulberry plantation management under future climate change scenarios.

## 1. Introduction

Ambient CO_2_ (aCO_2_) concentrations have increased from 280 ppm at the beginning of the industrial revolution to the current ~420 ppm, primarily due to anthropogenic activities such as extensive utilization of fossil fuels, deforestation, etc. According to the IPCC (2018), aCO_2_ could potentially reach to 550 ppm or higher by the end of this century, with significant implications for global climate patterns and plant responses [1]. Elevated CO_2_ (eCO_2_) contributes to temperature increases through the greenhouse effect, inducing spatially heterogeneous precipitation frequencies and patterns on a global scale, eventually leading to regional droughts under future climate change [2,3]. Additionally, eCO_2_ is commonly practiced in controlled environments such as greenhouses to enhance plant growth and productivity, making it relevant for both natural and agricultural systems. Drought stress typically affects various cellular and physiological processes, such as protein degradation, changes in membrane fluidity, and the inhibition of leaf photosynthesis, ultimately resulting in a decrease in crop yield by 25–30% and deteriorated quality [4,5,6]. However, it is worth noting that the growth-stimulating effect of eCO_2_ can partially offset the impacts caused by drought stress, particularly in C_3_ species [7,8,9]. Therefore, it is crucial to consider the abovementioned effects when assessing the overall impact of climate change on plant growth.

To comprehend the potential mechanism of agricultural production in response to eCO_2_ and drought stress, it is imperative to investigate alterations in the physiological processes and biochemical components of plants that are sensitive to both eCO_2_ and drought stress, such as leaf photosynthesis, chlorophyll fluorescence, soluble sugars, proline, and antioxidative ability [9,10,11,12]. Plant photosynthesis is a drought-sensitive process, and water deficit usually leads to a significant decline in leaf photosynthesis [5,10,13]. The decrease in leaf photosynthesis under water deficit may be associated with reduced stomatal conductance [14], nitrogen (N) and chlorophyll contents [15], and 1,5-bisphophate carboxylase/oxygenase (Rubisco) activity [16]. It has been observed that drought stress decreases the effective quantum yield of PSII photochemistry in wheat while also causing increased accumulation of proline and soluble sugar contents in barley and buckwheat [17,18]. Additionally, antioxidant enzymes such as superoxide dismutase (SOD), peroxidase (POD), and catalase (CAT) were found to be highly activated in soybean under drought stress in order to adapt and scavenge the free radicals induced by drought stress, accompanied by increased accumulation of malondialdehyde (MDA) [19,20]. Therefore, drought stress limits crop growth and grain yield by reducing aboveground biomass and modifying the shoot-to-root ratio through the development of deeper roots capable of acquiring more water [21].

The increase in atmospheric CO_2_ concentration, the primary C source for plants, exerts significant CO_2_ fertilization effects on crops [22,23,24] because the Rubisco enzyme in C_3_ plants is not saturated at the current atmospheric CO_2_ concentration [25,26]; thus, eCO_2_ can stimulate leaf photosynthesis and enhance plant growth and grain yield by facilitating C_3_ the uptake of more CO_2_ molecules by plants [27]. Previous studies have revealed that eCO_2_ can mitigate the adverse effects of drought stress on plant growth and physiological functioning by increasing the intercellular CO_2_ concentration (Ci), improving water use efficiency (WUE) while reducing transpiration rates [28,29,30,31]. A meta-analysis showed that eCO_2_ significantly increases both the net photosynthetic rate (37.2–46.3%) and WUE (84.2–88.9%) [9] while decreasing evapotranspiration by ~10% [32]. The increased WUE under eCO_2_ implies that plants may exhibit tolerance to future high CO_2_ concentrations under drought conditions [8,33]. Moreover, enhanced photosynthesis under eCO_2_ leads to an accumulation of carbohydrates under drought stress, resulting in increased cell osmotic potential and promoting osmotic adjustment [34]. Concurrently, the formation of reactive oxygen species (ROS) was also found to be mitigated under eCO_2_ due to increased electron consumption in C fixation processes [7,35]. However, the capacity of the CO_2_ fertilization effect to alleviate drought stress may vary not only among different plant species [36,37] but also depending on the levels of water deficit [30,38]. For instance, some studies have reported that eCO_2_ can effectively mitigate drought stress by enhancing WUE and photosynthesis in certain species, such as coffee [21] and soybean [19,33]. In contrast, other investigations have revealed that eCO_2_ did not significantly improve drought tolerance or even exacerbated water stress in species like wheat and sorghum, particularly under severe drought conditions [39,40]. These inconsistent findings may be attributed to differences in species-specific traits, experimental conditions, and the severity of the applied drought stress. For example, C_4_ plants, which have a different photosynthetic pathway, may not benefit as much from eCO_2_ as C₃ plants under drought stress [41,42]. Thus, there is currently no consistency regarding the specific physiological mechanisms by which the eCO_2_ concentration can affect plant growth and crop yield under drought stress.

In addition to its impact on plant physiology, drought also significantly affects the uptake, accumulation, and distribution of soil nutrients (particularly nitrogen (N), phosphorus (P), and potassium (K)). A recent meta-analysis revealed that drought stress generally decreases nutrient absorption from soil and leads to decreased N and P concentrations in plant tissue [43,44,45]. In contrast, eCO_2_ can alter the shoot-to-root ratio by stimulating deeper root growth, thereby enabling better water and nutrients uptake [9,30]. In general, plants under eCO_2_ store more C in their above- and below-ground biomass compared to those under aCO_2_ [35], leading to changes in their contents of N, P, and K and other nutrients in both plants and soil [46]. Meanwhile, eCO_2_ increases fluxes of organic compounds into soil due to a higher rate of plant litterfall and rhizodeposition, resulting in changes in soil organic carbon (SOC) content [47], the availability of soil nutrients [44], composition and activities of the microbial community [48,49], and activities of soil enzymes involved in C and N cycling [47,50]. Such changes in soil are predicted to result in stronger but uncertain feedback at the ecosystem level to eCO_2_. However, most studies have explored the individual effects of focusing on either eCO_2_ or drought stress on a plant–soil–microbe system, with limited studies addressing their combined effects on plant nutrient distribution and accumulation, soil nutrient availability, and enzyme activity.

Mulberry (*Morus alba* L.), a rapidly growing multipurpose cash plant, is extensively cultivated in southwest China, accounting for approximately 70% of China’s total plantations (~758,200 hectares) due to its significant economic value. However, this region is currently experiencing reduced precipitation and increased aridity, leading to a scarcity of freshwater resources for agricultural production [51]. These climatic changes pose a significant threat to mulberry cultivation, especially during prolonged drought periods [10,52]. Despite its sensitivity to extreme drought, mulberry exhibits remarkable adaptability to moderate environmental stressors, making it an ideal model for studying climate change adaptation [53,54]. Its high plasticity, deep root system, and efficient water use mechanisms enable it to thrive in arid and semi-arid regions, positioning it as a critical crop for sustainable agriculture in water-limited environments [55,56]. Additionally, mulberry leaves are the primary food source for silkworms, which are integral to the sericulture industry, a major economic driver in many developing regions [57]. The plant’s ability to produce high levels of biomass under moderate stress conditions, coupled with its role in carbon sequestration, further underscores its importance in mitigating climate change impacts [58]. Although studies have reported the morphological, physiological, and biochemical responses of mulberry plants to drought stress [10,52,59,60], most of these studies primarily focused on different mulberry cultivars and/or altered soil–water gradients without considering the potential effect of CO_2_. We previously observed a significant 33–40% increase in leaf biomass production of mulberry when the CO_2_ concentration was elevated from 410 to 710 ppm [61,62]. Leaf photosynthesis and WUE were enhanced by 550–800 ppm eCO_2_ in various mulberry cultivars, including Selection-13, Kanva-2, ‘Qinglong’ mulberry, Gui-sang-you 62, Qiangsang-1, and Nongsang-14, under well-watered conditions [61,62,63,64,65]. However, understanding regarding how eCO_2_ affects drought-induced changes in photosynthesis, PSII function, antioxidant defense mechanisms, and nutrient acquisition and partitioning, as well as soil enzyme activities. By understanding how mulberry responds to combined drought and eCO_2_ conditions, we can develop strategies to enhance its resilience and productivity, thereby supporting both economic and environmental sustainability. Here, we hypothesized that eCO_2_ might have a positive impact on mulberry performance under drought conditions. Specifically, the objectives were to investigate (1) the combined effects of eCO_2_ and drought stress on mulberry biomass accumulation; (2) the correlations between drought tolerance, physiological traits, and nutrient acquisition under eCO_2_; and (3) the potential mechanisms through which eCO_2_ could mitigate negative effects induced by drought stress via alterations in photosynthetic characteristics, antioxidant defense systems, nutrient uptake/distribution, and soil enzyme activities. Therefore, the following physiological traits of mulberry were investigated: leaf gas exchange, PSII efficiency, CAT, POD, SOD, MAD, and contents of proline and soluble sugar at both the leaf and root levels. Meanwhile, the uptake and partitioning of N, P, and K, along with soil enzyme activity. The anticipated findings will contribute to the development of effective water management strategies by elucidating the physiological responses of plants to future drought stress induced by elevated atmospheric CO_2_ levels.

## 2. Materials and Methods

### 2.1. Growth Chamber Experiments

This experiment was conducted in 12 environmentally controlled plant growth chambers that were located at the National Monitoring Base for Purple Soil Fertility and Fertilizer Efficiency (29°48′ N, 106°24′ E, 266.3 m above the sea level) on the campus of Southwest University, Chongqing, China. The rectangular structure of each chamber measures 1.5 × 1.0 × 2.5 m and is supported by a steel frame suspended 50 cm above a cement floor. The bottom floor of the growth chamber consists of polyvinyl chloride panels, while the four side walls and top roof are lined with by tempered glass (10 mm thick) with transparency allowing approximately 90% of natural sunlight to pass through. Humidity and temperature inside and outside the growth chamber were automatically maintained within ±0.5 °C air temperature and ±5% humidity using electronic control systems. When the humidity inside a chamber was higher than that outside, the inside air was pumped out using pump controlled by a mini-computer and filtered with solid anhydrous calcium chloride. The temperature was automatically maintained at 0.5 °C variation between inside and outside using an air conditioner controlled by a mini-computer. To maintain a targeted CO_2_ concentration (±30 ppm) inside each chamber, 90% pure CO_2_ gas was injected using a solenoid valve controlled by a mini-computer [42].

**Figure 1:**
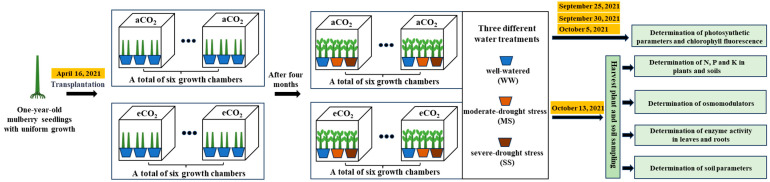
A schematic diagram showing the experimental designs and procedures. aCO_2_: ambient CO_2_ (420/470 ppm, day/night); eCO_2_: elevated CO_2_ (710/760 ppm).

The experiment was conducted using a split-plot design consisting two factors (CO_2_ and water or drought), with CO_2_ as the main factor and drought as the sub-factor. Based on previously observed daytime and night-time atmospheric CO_2_ concentrations at the study site, two different CO_2_ concentrations were applied: ambient CO_2_ (aCO_2_, 420 ppm daytime/470 ppm nighttime) for six growth chambers and eCO_2_ (710 ppm daytime/760 ppm nighttime) for other six growth chambers. Daytime was defined as 07:00 a.m. to 19:00 p.m., while nighttime was defined 19:00 p.m. to 07:00 a.m. The experimental designs and the procedures are illustrated in Figure 1.

One-year-old, uniformly growing mulberry seedlings (*Morus multicaulis* Perr. QiangSang-1) were used as experimental materials. Two seedlings were planted in a plastic pot (32 cm in diameter and 20 cm in height). The pots were filled with 11 kg air-dried soil (Eutric Regosol, FAO Soil Classification System). The soil was sieved (2 mm) and had a pH of 6.8, organic carbon content of 7.03 g kg^−1^, available N content of 31.21 mg kg^−1^, available P content of 11.47 mg kg^−1^, and available K content of 127 mg kg^−1^. Three pots were placed into each growth chamber. Mulberry seedlings were transplanted on 16 April 2021. During the first four months, all of the seedlings were regularly watered two or three times per week with 400 mL of tap water for each pot so that soil relative water content (RWC) was maintained at 75–85%. Meanwhile, 2.2 g N, 0.77 g P_2_O_5_, and 1.1 g K_2_O were applied per pot to meet the plants’ nutrient requirements.

After the four-month experimental period, three pots from each CO_2_ chamber were subjected to three different water treatments: well-watered (WW), moderate drought stress (MS), and severe drought stress (SS). Under the well-watered condition, plants were consistently irrigated as described above to maintain an RWC of 75–85%. Plants experiencing moderate drought stress received irrigation twice weekly with 200 mL of water per pot in order to maintain an RWC of 55–65%. Plants exposed to severe stress were irrigated twice weekly with 100 mL of water per pot to maintain an RWC of 35–45%. Soil volumetric water contents were measured using a soil moisture sensor (JXBS-3001, Qingdao, China), then converted to gravimetric water contents. Soil relative water content, which was calculated as the ratio of gravimetric water to saturated moisture content. These three water treatments were performed in each chamber; thus, there were six replicated chambers and six replicate pots for each CO_2_ concentration treatment. Additionally, pots were relocated among different chambers once fortnightly to minimize any potential effect of environmental variations across different growth chambers.

### 2.2. Determination of Photosynthetic Parameters

Measurements of photosynthetic parameters were conducted prior to harvest between 9:00 and 11:00 a.m. on sunny days, specifically on 25, 30 September and 5 October 2021 (data averaged for these three days). The third fully expanded leaf from the top was selected for determination of the net photosynthetic rate (Pn), stomatal conductance (gs), transpiration rate (E), and intercellular CO_2_ concentration (Ci) using a Li-6800 portable photosynthesis system (LICOR, Lincoln, NE, USA). Throughout the measurement, the saturated photosynthetically active radiation was maintained at 1600 μmolm^−2^ s ^−1^ using an integrated LED light source (Model 6800-01A, LI-COR Inc.) within the leaf chamber (Model 6800-02A Large Leaf Chamber, LI-COR Inc.). This light intensity was selected based on preliminary light-response curve experiments, which confirmed that it was sufficient to achieve light-saturated photosynthetic rates in the studied mulberry leaves [64]. The LED source provides uniform light distribution and stable intensity, ensuring accurate and reproducible measurements. Meanwhile, the leaf temperature in the leaf chamber was set at 25 ± 1 °C. For plants growing under eCO_2_ and aCO_2_, the leaf cavity’s CO_2_ concentration was maintained at 700 ppm and 420 ppm, respectively. The vapor-pressure deficit at the leaf surface was consistently maintained at 1.2 ± 0.2 kPa. Water use efficiency (WUE) was calculated as the ratio of Pn to E. The intercellular CO_2_ and ambient CO_2_ concentrations were used to the calculate Ci/Ca ratio.

### 2.3. Determination of Chlorophyll Fluorescence

The chlorophyll fluorescence was also determined using the Li-6800 fluorescence leaf chamber (LI-COR, Lincoln, USA) connected to an Li-6800 portable photosynthesis system on the same leaves used for photosynthetic leaf gas exchange measurements on 25 and 30 September and 5 October 2021 (data averaged for these three days). To ensure that all photosystem-II (PS-II) reaction centers were open, the leaves were wrapped with tin foil and darkened for 30 min. The following parameters were measured: initial fluorescence yield (F0) and maximum fluorescence yield (Fm) in the dark-adapted stage, minimum fluorescence yield (F0’) and maximum fluorescence (Fm′) in the light-adapted stage, and steady-stage fluorescence yield (Fs). Subsequently, the effective quantum yield of PSII photochemistry (Φ_PSII_), maximal quantum yield of PSII photochemistry (Fv/Fm), actual photosynthetic efficiency of PSII under illumination (Fv′/Fm′), photochemical quenching coefficient (qP), non-photochemical quenching coefficient (NPQ), and PSII electron transport rate (ETR) were calculated using the aforementioned measured parameters. The calculation formulas are expressed as follows: Φ_PSII_ = (Fm′ − Fs)/Fm′, NPQ = (Fm − Fm′)/Fm′, qP = (Fm′ − Fs)/(Fm′ − F0’), and ETR = 0.8 × Φ_PSII_ × PPFD [66].

### 2.4. Plant and Soil Sampling

The plants were harvested on 13 October 2021, and the leaves used to measure photosynthetic parameters were collected, immediately frozen in liquid nitrogen, and subsequently stored in a −80 °C freezer for determination of enzyme activity and osmomodulators. The plant tissues were divided into leaves, stems, and roots. The fresh roots were thoroughly washed with tap water, followed by rinsing with deionized water. A portion of the fresh roots was immediately frozen in liquid nitrogen after collection and stored at −80 °C until analysis of enzyme activity and osmomodulators. The remaining portion of the roots, leaves, and stems was dried at 105 °C for 30 min initially, then further dried at 75 °C for ≥ 48 h until reaching a constant weight to determine the biomass and N, P, K, etc. Soil samples from each growth pot were collected after thorough mixing and removal of debris and fine roots and subsequently air-dried (2 mm sieved) to determine their chemical properties and enzyme activities.

### 2.5. Determination of N, P, and K in Plants and Soils

The dried leaf, stem, and roots of each sample were finely ground into a powder before being subjected to digestion with 98% sulfuric acid and 30% hydrogen peroxide. Tissue N, P, and K concentrations were determined by the trace Kjeldahl method, vanadium molybdate yellow colorimetry, and flame photometry [67], respectively. Soil available N (AN) was measured using the micro-diffusion technique after alkaline hydrolysis. Soil available P (AP) was extracted with 0.5 M NaHCO_3_, followed by measurement using the Mo-Sb anti spectrophotometric method. Soil available K (AK) was extracted with 1.0 M ammonium acetate, then determined by flame photometry [67].

### 2.6. Determination of Osmomodulators

The soluble sugars were determined using anthrone colorimetry [57]. Briefly, 0.5 g of fresh samples was extracted with 10 mL H_2_O in boiling water for 20 min. The extraction was mixed with 0.5 mL of anthrone ethyl acetate and 5 mL of sulfuric acid, followed by incubation in boiling water for 10 min. The absorbance was measured spectrophotometrically at a wavelength of 620 nm.

The determination of proline was conducted using the sulfosalicylic acid method [68]. Briefly, 0.5 g of fresh sample was extracted with 5 mL of 3% sulfosalicylic acid in boiling water for 10 min. Then, 2 mL of filtrate was mixed with 2 mL glacial acetic acid and 2 mL of acid ninhydrin. The mixture was then subjected to a boiling water bath for 30 min. After cooling, 4 mL toluene was added into the mixture. The absorbance was measured at a wavelength of 520 nm.

MDA was determined using the thiobarbituric acid (TAB) method [68]. Briefly, 0.5 g of fresh sample was homogenized with 5 mL of 5% TAB solution. Then, 2 mL of the extracted solution was mixed with 2 mL of 0.67% TAB solution and boiled for 15 min. The absorbance of the incubated supernatant was spectrophotometrically measured at wavelengths of 450 nm, 532 nm, and 600 nm.

### 2.7. Determination of Enzyme Activity in Leaves and Roots

The activity of POD was determined using the guaiacol method and expressed in terms of enzyme units per gram fresh weight (U g^−1^ FW) [68]. Briefly, 0.5 g of fresh sample was extracted with 10 mL of 0.1 mM phosphate buffer (PBS, pH 6.0), followed by centrifugation at 4000 rpm for 15 min. The supernatant was collected in a 100 mL volumetric flask. Subsequently, the reaction mixture contained 1 mL of enzyme extraction, 2.5 mL guaiacol (0.1 M), and 0.5 mL 30% H_2_O_2_. The change in absorbance at 470 nm, measured as 1.0 per min, was considered one unit of POD activity.

The activity of SOD was determined using the nitrogen blue tetrazolium (NBT) assay method [68]. Briefly, 0.5 g of fresh sample was extracted with 10 mL of 0.05 mM PBS (pH 7.8), then centrifuged at 10,000 rpm for 10 min. The supernatant was collected in a 100 mL volumetric flask. Subsequently, the reaction mixture contained 0.3 mL of enzyme extraction, 1.5 mL of 0.05 mM PBS, 0.3 mL of 130 mM Met solution, 0.3 mL 750 µM NBT solution, 0.3 mL 100 µM EDTA-Na_2_ solution, and 0.3 mL 20 µM riboflavin. The reaction mixture was exposed to fluorescence under 4000 luxes for 20 min; then, the absorbance was measured at a wavelength of 560 nm. The SOD activity was defined as the amount of enzyme required to inhibit 50% of NBT reduction per g fresh weight (U g^−1^ FW).

The activity of CAT was determined by potassium permanganate titration [57]. Briefly, 0.5 g of fresh sample was extracted with 10 mL of 0.2 mM PBS (pH 7.0), then centrifuged at 4000 rpm for 10 min. The supernatant was collected in a 100 mL volumetric flask. Subsequently, 5 mL of the extraction was added to 2.5 mL of 0.1 M H_2_O_2_ and placed in a 30 °C water bath for 10 min; then 2.5 mL of 10% sulfuric acid was added, and the mixture was titrated with 0.1 M KMnO_4_ until a pink color was achieved, followed by 30 s without color change. CAT activity is expressed as mg of hydrogen peroxide decomposed per minute per g fresh weight of sample.

### 2.8. Determination of Soil Enzyme Activity

The soil invertase activity was determined using the 3,5-dinitrosalicylic acid colorimetry method [69]. Briefly, 1.0 g of air-dried soil was incubated with a mixture containing 0.2 of ml toluene, 3 mL of 8% (*w*/*v*) sucrose, and 1 mL of PBS (pH 5.5) at 37 °C for 24 h. After incubation, 1 mL extraction with 3 mL 3,5-dinitrosalicylic acid was incubated in boiling water for 5 min. Subsequently, the absorbance was measured at a wavelength of 508 nm. Results are expressed as mg of glucose released per g dry soil per h.

The soil urease activity was determined using sodium phenol–sodium hypochlorite colorimetry [69]. Briefly, 1.0 g of air-dried soil was incubated with 0.2 mL of toluene, 2 mL of 10% urea solution, and 4 mL of citrate buffer (pH 6.7) at 37 °C for 24 h. After incubation, the reaction mixture contained 1 mL enzyme extraction, 4 mL sodium phenol solution, and 3 mL sodium hypochlorite solution. The absorbance of the reaction solution was measured at a wavelength of 578 nm. Results are expressed as mg of NH_4_^+^–N released per g soil per h.

The neutral phosphatase activity was determined using disodium benzene phosphate colorimetry [69]. Briefly, 1.0 g of air-dried soil was incubated with 0.2 mL of toluene and 5 mL of 0.5% disodium phenyl phosphate solution for 24 h at 37 °C. After incubation, 1 mL extraction with 0.5 mL of citrate buffer (pH 7.0), 1 mL of 8% potassium ferrocyanide (*w*/*v*), and 1 mL of 2% 4-aminoantipyrine were thoroughly mixed. Subsequently, the absorbance of the reaction solution was measured at a wavelength of 510 nm. Results are expressed as mg of phenol per g soil per 4 h.

### 2.9. Statistical Analysis

Statistical analysis was performed using SPSS 19.0 software (SPSS Inc., Chicago, IL, USA). Results (means ± SE, *n* = 6) between treatments were analyzed by two-way analysis of variance (ANOVA), and significant differences were compared with the Duncan multiple range test at *p* < 0.05 unless mentioned otherwise. Structural equation modeling (SEM) was employed in the Amos 21.0 software package to investigate potential causative relationships among measured factors (Smallwaters Corporation, Chicago, IL, USA). Figures were generated using Origin Pro 2018 software (OriginLab Corp., Northampton, MA, USA).

## 3. Results

### 3.1. Effects of eCO_2_ and Drought on Photosynthetic Parameters of Leaves of Mulberry Plants

**Figure 2:**
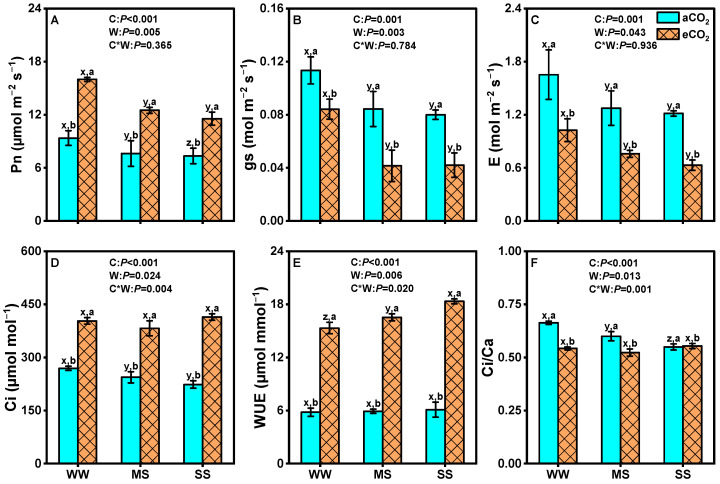
Net photosynthetic rate (Pn, **A**), stomatal conductance (gs, **B**), transpiration rate (E, **C**), intercellular CO_2_ (Ci, **D**), leaf water use efficiency (WUE, **E**), and the ratio of Ci to ambient CO_2_ (Ci/Ca, **F**) of the fully expanded upper leaf of 18-month-old mulberry plants under ambient CO_2_ (aCO_2_, 420/470 ppm, day/night), elevated CO_2_ (eCO_2_, 710/760 ppm), and varying water regimes (well-watered, WW; moderate drought stress, MS; severe drought stress, SS). Data (means ± SE, *n* = 6) followed by different letters indicate significant differences between CO_2_ treatments for the same water regime (a, b) and among water treatments for the same CO_2_ concentration (x, y, z) at *p* < 0.05. Statistical comparisons (two-way ANOVA) between water regimes and CO_2_ treatments, as well as their interaction (water × CO_2_), are presented for each variable.

Compared to well-watered conditions, drought stress significantly decreased the net photosynthetic rates, stomatal conductance, transpiration rates, intercellular CO_2_ concentration, and Ci/Ca ratio of mulberry plants, regardless of CO_2_ concentrations (Figure 2). However, eCO_2_ significantly mitigated these effects. Under eCO_2_, the net photosynthetic rates increased by 27%, 25%, and 37% (*p* = 0.001, Figure 2A), while stomatal conductance decreased by 38%, 41%, and 48% (*p* = 0.001, Figure 2B) and leaf transpiration rates decreased by 26%, 51%, and 48% (*p* = 0.001, Figure 2C) under well-watered conditions, moderate drought, and severe drought stress, respectively. As a result, leaf WUE was dramatically enhanced by 96%, 104%, and 163%, respectively (*p* = 0.001, Figure 2E), under the same conditions. Meanwhile, mulberry plants under eCO_2_ exhibited higher Ci values than those grown under aCO_2_, regardless of soil water status (Figure 2D). Nevertheless, the Ci/Ca ratio was significantly lower under eCO_2_ than under aCO_2_ under both well-watered and moderate-drought-stress conditions (Figure 2F). Two-way ANOVA revealed significant interaction effects between eCO_2_ and drought on leaf WUE (*p* = 0.020), Ci (*p* = 0.004) and Ci/Ca ratio (*p* = 0.001) but not on the net photosynthetic rate (*p* = 0.37), stomatal conductance (*p* = 0.78), or transpiration rate (*p* = 0.94) (Figure 2).

### 3.2. Effects of eCO_2_ and Drought on Leaf Chlorophyll Fluorescence of Mulberry Plants

**Figure 3:**
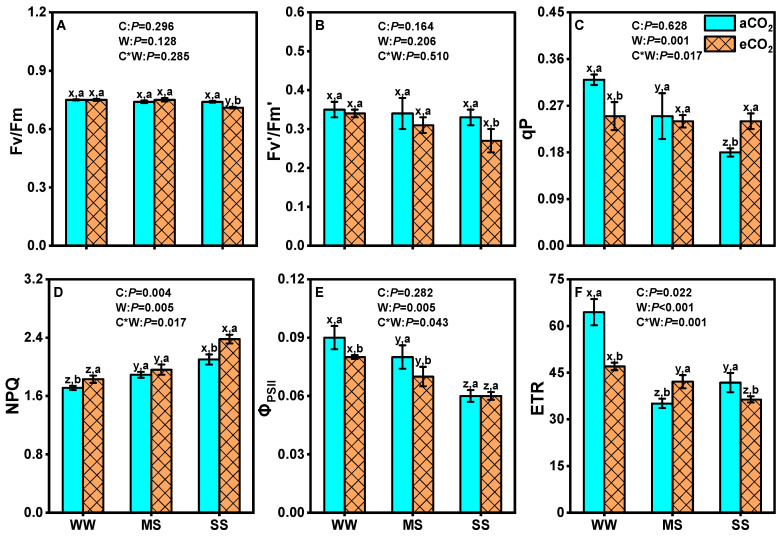
Fv/Fm (**A**), Fv′/Fm′ (**B**), qP (**C**), NPQ (**D**), *Φ_PSII_* (**E**) and ETR (**F**) of the fully expanded upper leaf of mulberry plants under ambient CO_2_ (aCO_2_, 420/470 ppm, day/night), elevated CO_2_ (eCO_2_, 710/760 ppm), and varying water regimes (well-watered, WW; moderate drought stress, MS; severe drought stress, SS). Data (means ± SE, *n* = 6) followed by different letters indicate significant differences between CO_2_ treatments for the same water regime (a, b) and among water treatments for the same CO_2_ concentration (x, y, z) at *p* < 0.05. Statistical comparisons (two-way ANOVA) between water regimes and CO_2_ treatments, as well as their interaction (water × CO_2_), are presented for each variable.

Compared to well-watered conditions, drought stress significantly decreased *Φ_PSII_* and ETR under eCO_2_ and aCO_2_, as well as qP under aCO_2_, while increasing NPQ, regardless of CO_2_ concentrations (Figure 3). The qP was 21.9% lower in eCO_2_ than in aCO_2_ under well-watered conditions but 33.3% higher under severe drought (Figure 3C). Similarly, eCO_2_ also decreased *Φ_PSII_* by 11.1% under both well-watered and moderate-drought conditions (Figure 3E) and decreased the ETR by 27.0% and 12.9% under well-watered and severe-drought conditions, respectively (Figure 3F). Conversely, eCO_2_ significantly increased NPQ by 7.1%, 4.1%, and 13.4% under well-watered, moderate-drought, and severe-drought conditions, respectively (Figure 3D). Neither Fv/Fm nor Fv′/Fm′ was affected by eCO_2_ or drought stress (*p* > 0.05, Figure 3A,B). Two-way ANOVA revealed significant interaction effects between eCO_2_ and drought on qP (*p* = 0.001), NPQ (*p* < 0.05), *Φ_PSII_* (*p* < 0.05) and ETR (*p* = 0.001) but not on Fv/Fm (*p* > 0.285) or Fv′/Fm′ (*p* > 0. 5, Figure 3).

### 3.3. Effects of eCO_2_ and Drought on Osmotic Regulators of Mulberry Plants

**Figure 4:**
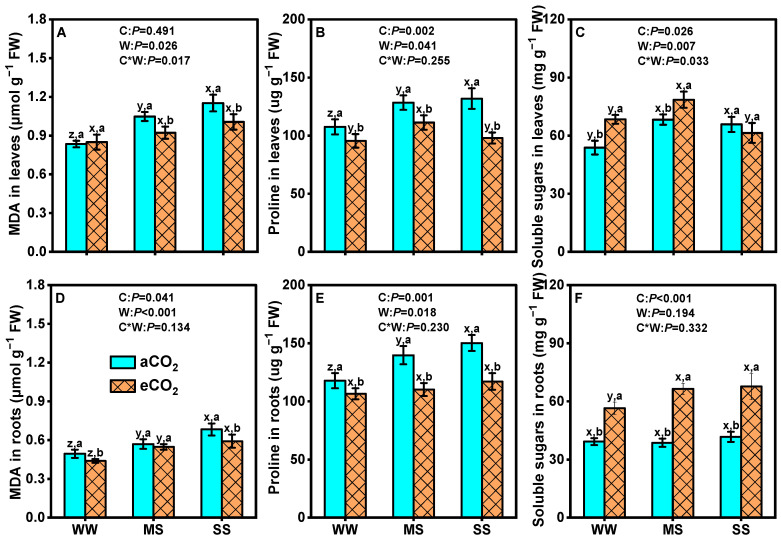
Malondialdehyde (MDA, **A**,**D**), proline (**B**,**E**), and soluble sugar (**C**,**F**) in leaves and roots of mulberry plants under ambient CO_2_ (aCO_2_, 420/470 ppm, day/night), elevated CO_2_ (eCO_2_, 710/760 ppm), and varying water regimes (well-watered, WW; moderate drought stress, MS; severe drought stress, SS). Data (means ± SE, *n* = 6) followed by different letters indicate significant differences between CO_2_ treatments for the same water regime (a, b) and among water treatments for the same CO_2_ concentration (x, y, z) at *p* < 0.05. Statistical comparisons (two-way ANOVA) between water regimes and CO_2_ treatments, as well as their interaction (water × CO_2_), are presented for each variable.

In comparison to well-watered conditions, leaf MDA concentration was substantially increased by 25.0% and 38.1% under moderate and severe stresses at aCO_2_ (*p* < 0.05, Figure 4A), while no significant changes were observed ateCO_2_ (*p* > 0.05, Figure 4A). Drought stress significantly increased root MDA, irrespective of CO_2_ concentrations (*p* < 0.001, Figure 4D). In contrast, eCO_2_ decreased leaf MDA by 12.4% and 13.0% under moderate and severe stresses, respectively, and root MDA by 10.2% and 13.2% under well-watered and severe stress conditions, respectively (*p* < 0.05, Figure 4D). Similarly, eCO_2_ significantly reduced proline concentrations in leaves by 11.2%, 13.3%, and 26.7% and in roots by 9.4%, 21.2%, and 22.0% under well-watered, moderate stress, and severe stress conditions, respectively. However, both leaf and root proline concentrations were obviously increased with declining soil water under aCO_2_ (*p* < 0.05, Figure 4B,E). Conversely, eCO_2_ significantly enhanced soluble sugar concentrations in leaves by 27.1%, 15.0%, and 6.7% and in roots by 43.9%, 71.8%, and 62.3% under well-watered, moderate stress, and severe stress conditions, respectively (*p* < 0.05, Figure 4C,E). Two-way ANOVA results showed significant interaction effects between eCO_2_ and drought on leaf soluble sugar (*p* = 0.03) and leaf malondialdehyde (*p* = 0.02) but not on root soluble sugar (*p* = 0.33), root malondialdehyde (*p* = 0.13), leaf proline (*p* = 0.23), or root proline (*p* = 0.23, Figure 4).

### 3.4. Effects of eCO_2_ and Drought on Antioxidant Enzyme Activities in Mulberry Plants

**Figure 5:**
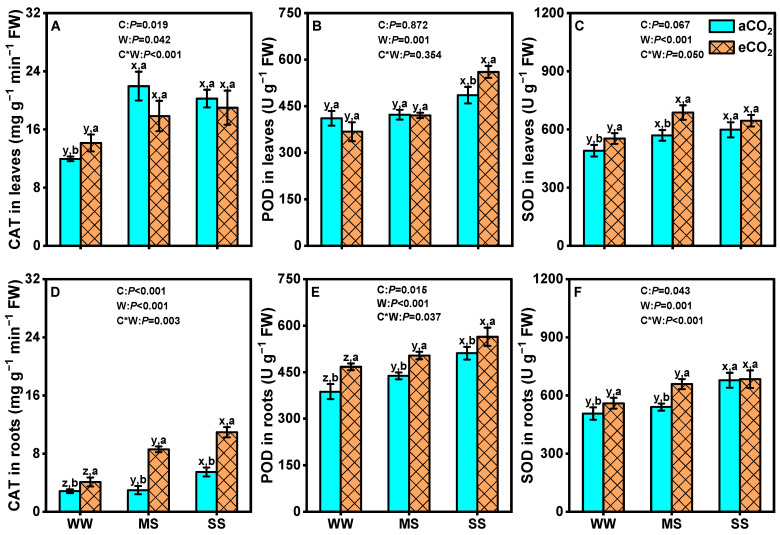
Catalase (CAT: **A**,**D**), peroxidase (POD: **B**,**E**), and superoxide dismutase (SOD: **C**,**F**) activities in leaves and roots of 18-month-old mulberry plants under ambient CO_2_ (aCO_2_, 420/470 ppm, day/night), elevated CO_2_ (eCO_2_, 710/760 ppm), and varying water regimes (well-watered, WW; moderate drought stress, MS; severe drought stress, SS). Data (means ± SE, *n* = 6) followed by different letters indicate significant differences between CO_2_ treatments for the same water regime (a, b) and among water treatments for the same CO_2_ concentration (x, y, z) at *p* < 0.05. Statistical comparisons (two-way ANOVA) between water regimes and CO_2_ treatments, as well as their interaction (water × CO_2_), are presented for each variable.

The activities of CAT, POD, and SOD in both leaves and roots consistently increased as soil water declined under both aCO_2_ and eCO_2_ (Figure 5). Elevated CO_2_ significantly increased CAT activity in leaves by 18.3% under well-watered conditions, and in roots by 44.5%, 188.2%, and 100.4% under well-watered, moderate-drought, and severe-drought conditions, respectively (*p* < 0.05, Figure 5A,D). Similarly, POD activity increased in leaves by 15.5% under severe drought, and in roots by 20.6%, 12.4%, and 10.3% under well-watered, moderate-drought, and severe-drought conditions, respectively (*p* < 0.05, Figure 5E). SOD activity exhibited a similar pattern, with higher activity under eCO_2_ compared to under aCO_2_ under well-watered and moderate-drought conditions, but no significant changes were observed under severe-drought (*p* < 0.05, Figure 5C,F). Two-way ANOVA results showed a significant interaction effect between eCO_2_ and drought on CAT activity in leaves (*p* < 0.001) and roots (*p* = 0.003), POD activity in roots (*p* = 0.037), and SOD activity in leaves (*p* = 0.050) and roots (*p* < 0.001) but not on POD activity in leaves (*p* = 0.354) (Figure 5).

### 3.5. Effects of eCO_2_ and Drought on Tissue N, P, and K in Mulberry Plants

Compared to well-watered conditions, drought stress significantly decreased the stem N concentration under both aCO_2_ and eCO_2_, as well as the root N concentration under aCO_2_, but increased the leaf N concentration under aCO_2_ (Table 1). Under eCO_2_, leaf N decreased by 6.1%, 10.1%, and 13.0% (*p* = 0.001) and stem N decreased by 10.5%, 15.9%, and 2.2% (*p* = 0.042) under well-watered, moderate-drought, and severe-drought conditions, respectively (Table 1), while root N remained unaffected (*p* > 0.05, Table 1).

Similarly, P concentrations in leaves, stems, and roots were consistently lower under eCO_2_ than under aCO_2_ across all water treatments (all *p* < 0.05, Table 1), except for stem P under severe drought and root P under moderate drought, which showed no significant differences. Compared to the well-watered treatment, moderate drought and severe drought reduced stem P by 15.1% and 13.1%, respectively, under aCO_2_, and by 14.7% and 0.8%, respectively, under eCO_2_. Conversely, the root P concentration increased significantly under moderate drought and severe drought under eCO_2_ but not under aCO_2_ (*p* = 0.044; Table 1).

In addition, only the leaf K concentration exhibited a consistent increase with declining soil water, regardless of CO_2_ conditions (*p* = 0.003; Table 1). In contrast, eCO_2_ had no significant effect on K concentrations in leaves, stems, or roots (all *p* > 0.05, Table 1). Two-way ANOVA revealed significant interactions between eCO_2_ and drought for both N and P concentrations in roots (*p* < 0.05; Table 1).

### 3.6. Effects of eCO_2_ and Drought on Tissue Accumulations and Allocation of N, P, and K in Mulberry Plants

**Figure 6:**
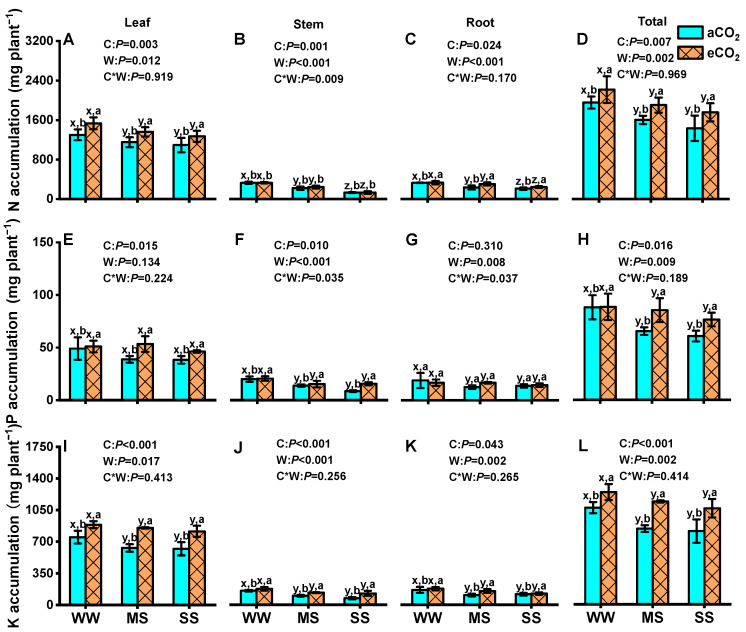
Accumulation of nitrogen (N: **A**–**D**), phosphorus (P: **E**–**H**), and potassium (K: **I**–**L**) in leaves, stems, roots, and in total plants in 18-month-old mulberry under ambient CO_2_ (aCO_2_, 420/470 ppm, day/night), elevated CO_2_ (eCO_2_, 710/760 ppm), and varying water regimes (well-watered, WW; moderate drought stress, MS; severe drought stress, SS). Data (means ± SE, *n* = 6) followed by different letters indicate significant differences between CO_2_ treatments for the same water regime (a, b) and among water treatments for the same CO_2_ concentration (x, y, z) at *p* < 0.05. Statistical comparisons (two-way ANOVA) between water regimes and CO_2_ treatments, as well as their interaction (water × CO_2_), are presented for each variable.

In general, drought stress significantly decreased the accumulation of N, P, and K in the leaves, stems, roots, and total plant under both aCO_2_ and eCO_2_, except for leaf P accumulation (Figure 6). Severe drought resulted in reductions in N, P, and K accumulation in the total plant of 26.7%, 31.0%, and 24.3% under aCO_2_ and of 20.7%, 13.5%, and 14.4% under eCO_2_, respectively. Nevertheless, eCO_2_ significantly increased N, P, and K accumulation in leaves, stems, roots, and the total plant subjected to well-watered, moderate-drought, and severe-drought conditions. Compared to aCO_2_, eCO_2_ significantly increased total-plant N accumulation by 13.3%, 18.8%, and 22.6% (*p* = 0.007, Figure 6D) and total-plant K accumulation by 16.1%, 36.0%, and 31.3% under well-watered, moderate-drought, and severe-drought conditions, respectively (*p* < 0.001, Figure 6L). Similarly, eCO_2_ also increased total-plant P accumulation by 30.7% and 25.9% under moderate drought and severe drought, respectively (*p* = 0.016), but had no significant effect under well-watered conditions. Two-way ANOVA revealed significant interaction effects between eCO_2_ and drought on stem N and stem and root P accumulation (*p* < 0.05, Figure 6).

**Figure 7:**
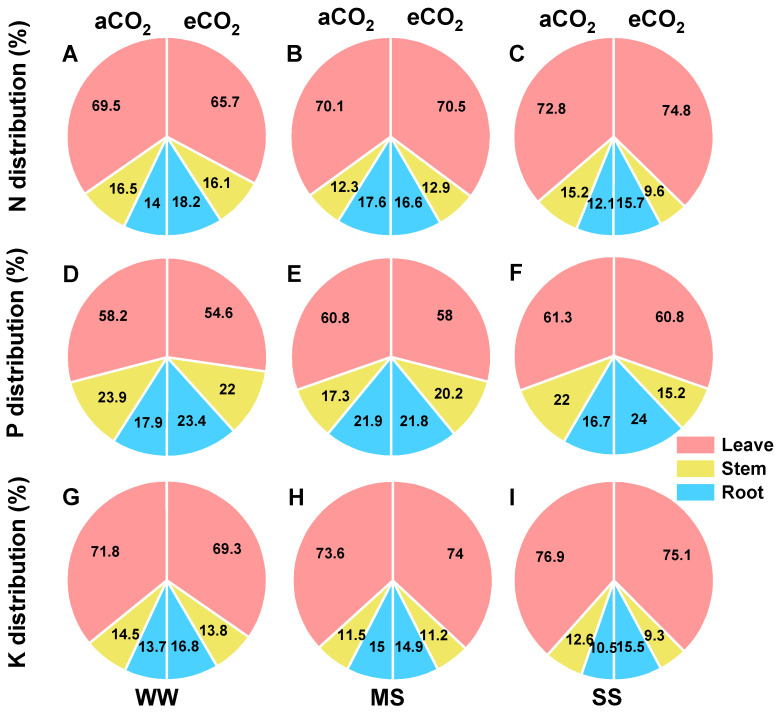
Leaf, stem, and root distribution of nitrogen (N: **A**–**C**), phosphorus (P: **D**–**F**), and potassium (K: **G**–**I**) in mulberry plants under ambient CO_2_ (aCO_2_, 420/470 ppm, day/night), elevated CO_2_ (eCO_2_, 710/760 ppm), and varying water regimes (well-watered, WW; moderate drought stress, MS; severe drought stress, SS).

Furthermore, severe drought significantly enhanced the allocations of N, P, and K to the leaves but decreased their allocations to the stems and roots of mulberry, regardless of CO_2_ concentration. Conversely, eCO_2_ decreased N, P, and K allocations to the leaves and stems but increased their allocations to roots under well-watered and severe-drought conditions (Figure 7). 

### 3.7. Effects of eCO_2_ and Drought on Soil Nutrients and Soil Enzyme Activities

**Figure 8:**
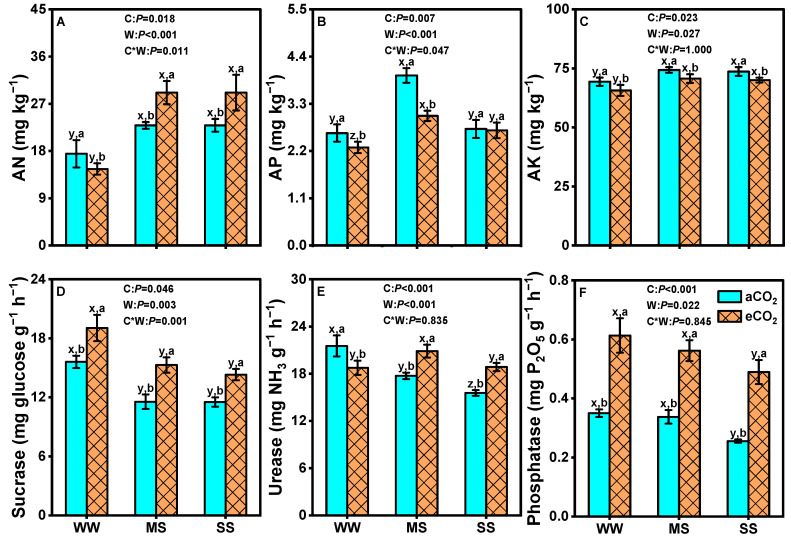
Available nitrogen (AN, **A**), available phosphorus (AP, **B**), available potassium (AK, **C**) in soil, as well as soil sucrase (**D**), urease (**E**), and phosphatase (**F**) activities of mulberry plants, under ambient CO_2_ (aCO_2_, 420/470 ppm, day/night), elevated CO_2_ (eCO_2_, 710/760 ppm), and varying water regimes (well-watered, WW; moderate drought stress, MS; severe drought stress, SS). Data (means ± SE, *n* = 6) followed by different letters indicate significant differences between CO_2_ treatments for the same water regime (a, b) and among water treatments for the same CO_2_ concentration (x, y, z) at *p* < 0.05. Statistical comparisons (two-way ANOVA) between water regimes and CO_2_ treatments, as well as their interaction (water × CO_2_), are presented for each variable.

The response of soil AN, AP, and AK to decreasing soil water status exhibited an increasing trend under both aCO_2_ and eCO_2_ (Figure 8). Soil AN showed an asymmetric response to CO_2_ concentration along the soil water gradient, where it was significantly lower under eCO_2_ than under aCO_2_ under the well-watered condition but higher under eCO_2_ than under aCO_2_ under moderate-drought and severe-drought conditions (*p* = 0.018, Figure 8A). Soil AP and AK were significantly lower under eCO_2_ than under aCO_2_ along the soil water gradient (*p <* 0.05, Figure 8B,C).

Moreover, regardless of soil water status, eCO_2_ generally significantly increased the activities of soil sucrase and phosphatase, although these enzyme activities obviously decreased with declining soil water content under both aCO_2_ and eCO_2_ (*p <* 0.05, Figure 8D,F). In contrast to the observed trends in soil sucrase and phosphatase activities, urease activity exhibited a different pattern: it decreased along the soil water gradient under aCO_2_ but slightly increased under moderate-drought conditions under eCO_2_ (*p <* 0.05, Figure 8E). Two-way ANOVA showed significant interaction effects between eCO_2_ and drought on soil AN (*p* = 0.023), AP (*p* = 0.027), and sucrase (*p* < 0.001, Figure 8).

### 3.8. Effects of eCO_2_ and Drought on Biomass Production in Mulberry Plants

Compared to the well-watered treatment, drought stress significantly decreased the biomass production in leaves, stems, roots, and total plants, regardless of CO_2_ concentration. Moderate drought and severe drought resulted 14.7% and 31.8% reductions in total biomass production, respectively, under aCO_2_ and 11.3% and 22.8% reductions, respectively, under eCO_2_. Conversely, a very strong effect of CO_2_ fertilization on mulberry growth was observed under both well-watered and drought stress conditions, with the effect more pronounced as soil water status declined. Specifically, eCO_2_ increased the leaf, stem, root, and total plant biomass production by 26.1%, 20.1%, 20.4%, and 23.1%, respectively, under well-watered conditions; by 32.0%, 29.4%, 18.8%, and 28.0%, respectively, under moderate drought; and by 35.7%, 83.7%, 15.3%, and 39.2% (all *p* ≤ 0.01), respectively, under severe drought (Table 2). Additionally, eCO_2_ significantly decreased the root/shoot ratio under severe drought. Two-way ANOVA showed a significant interaction effect between eCO_2_ and drought on stem biomass production only (*p* = 0.037, Table 2).

### 3.9. Multivariate Analyses

**Figure 9:**
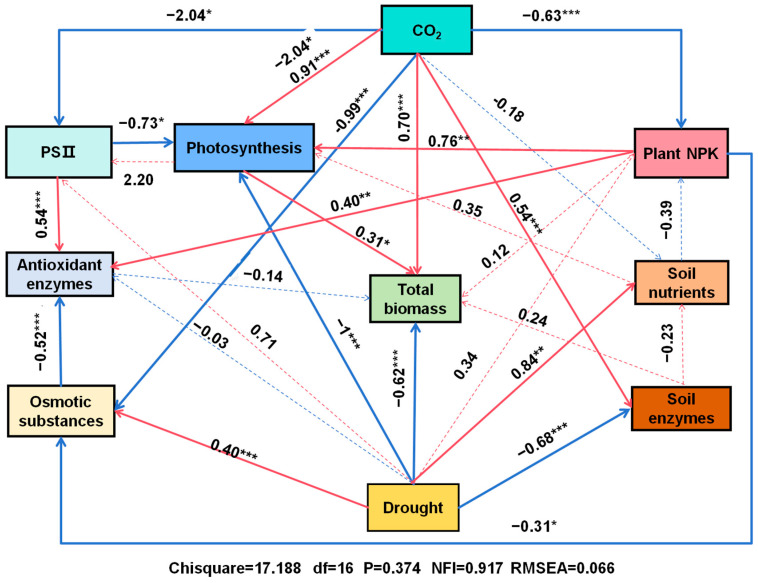
Structural equation model (SEM) showing relationships among CO_2_ concentration; soil water regime; photosynthetic parameters; PSII efficiency; antioxidant enzyme activities; osmotic substance concentrations; N, P, and K concentrations; soil nutrients; soil enzyme activities; and total biomass production of mulberry plants. Numbers above arrows are path coefficients. Bold and dashed lines indicate significant (*p* < 0.05) and non-significant (*p* > 0.05) paths, respectively. *, ** and *** significant at *p ≤* 0.05, *p ≤* 0.01 and *p ≤* 0.001, respectively.

To explore the potential mechanisms underlying the impact of eCO_2_ on plant response to drought stress, particularly in terms of changes in biomass production, we employed structural equation modeling (SEM) to analyze the relationships between eCO_2_ concentration, drought treatments, gas exchange, chlorophyll fluorescence, osmotic regulators, antioxidant enzyme activities, plant nutrient uptake, soil properties, and biomass production. The SEM analysis showed that drought had significant direct, positive effects on osmotic substances and soil nutrients while exerting significant direct, negative effects on photosynthesis, total biomass production, and soil enzyme activities. In contrast, eCO_2_ had significant direct, positive effects on photosynthesis, total biomass, and soil enzyme activities and significant direct, negative effects on chlorophyll fluorescence; osmotic substances; and plant N, P, and K (Figure 9).

## 4. Discussion

Mulberry plants growing in the majority of southwest China frequently experience a moderate drought due to a progressive reduction in soil water availability [10,70,71]. Drought stress has been widely reported to significantly reduce both photosynthesis and biomass accumulation in mulberry plants [72], in agreement with our study findings (Figure 2A, Table 2). In addition, mulberry production faces future challenges due to increases in , drought frequency, and atmospheric CO_2_ concentration. This study expands upon previous knowledge regarding how mulberry plants can cope with water scarcity and discusses the dynamics through which eCO_2_ mitigates the effects of prolonged drought stress by enhancing CO_2_ uptake and biomass production, altering nutrient uptake and partitioning while decreasing oxidative pressure.

### 4.1. eCO_2_ Alleviates Adverse Effects of Drought on Plant Biomass Production and Photosynthesis

Under drought conditions, 710 ppm eCO_2_ significantly stimulated the above- and below-ground biomass of mulberry, resulting in a substantial mitigation effect on mulberry productivity (Table 2). This finding agrees with previous studies conducted on coffee plants, *Picea abies*, *Pinus halepensis*, and *Populus tremula* under 700–867 ppm eCO_2_ in open-top chambers or free-air CO_2_ enrichment experiments [7,30,73,74]. The increase in plant biomass production due to eCO_2_ can be primarily attributed to biochemical and photochemical processes such as photosynthesis and respiration [75,76]. Specifically, plants grown under eCO_2_ typically exhibit a 30–60% greater leaf photosynthetic rate due to increased Rubisco carboxylation while competitively suppressing plant photorespiration and dark respiration [41,77]. In this study, eCO_2_ directly stimulated photosynthesis in mulberry through increased CO_2_ uptake (Figure 2A). A considerably greater intercellular CO_2_ concentration was observed under eCO_2_ (Figure 2D), indicating that the carboxylation rate of Rubisco was constrained by CO_2_ diffusion into the carboxylation sites under aCO_2_. Consequently, eCO_2_ favored the carboxylation reaction of Rubisco over its oxygenation reaction (Figure 5), thereby enhancing carbon assimilation by increasing Ci for Rubisco carboxylation. These results are consistent with findings reported by Kelly et al. (2019) [78] and Fan et al. (2020) [38]. The enhanced photosynthetic rates under eCO_2_ directly contribute to higher carbon assimilation, which is a key driver of biomass accumulation and overall productivity in mulberry. This is particularly evident in the significant increase in above- and below-ground biomass observed in our study (Table 2). The greater carbon fixation under eCO_2_ provides more resources for growth and development, enabling mulberry to maintain productivity, even under drought stress. Additionally, the increased carbon assimilation not only supports biomass production but also enhances the plant’s ability to cope with drought stress. By maintaining higher photosynthetic rates, mulberry can accumulate more carbohydrates, which are essential for osmotic adjustment and energy metabolism under water-limited conditions [79]. This explains the mitigation of drought stress observed in our study.

Despite the significant impact of eCO_2_ on leaf photosynthesis, its effects may be partially mitigated by drought stress. This is evidenced by a greater decline in net photosynthetic rate observed in plants experiencing moderate drought and severe drought compared to well-watered plants (Figure 2A). Such a reduction in the net photosynthetic rate under drought stress could be attributed to both diffusive limitations (such as gs and Ci, as shown in Figure 2B,D) and non-diffusive limitations related to biochemical processes [80]. These observations were supported by decreased leaf N, P, and K levels and their correlations with leaf photosynthesis in mulberry plants (Figure 9). Despite the partial mitigation of photosynthetic rates under drought stress, the overall enhancement of carbon assimilation under eCO_2_ still plays a critical role in sustaining mulberry growth and productivity. The ability of mulberry to maintain higher photosynthetic rates under combined eCO_2_ and drought conditions highlights its potential as a resilient crop for sustainable agriculture in regions affected by climate change.

However, it is important to critically assess potential limitations and trade-offs associated with long-term exposure to eCO_2_. One potential limitation is the acclimation response, where plants may exhibit reduced photosynthetic efficiency over time due to downregulation of Rubisco and other photosynthetic enzymes [62,81]. This acclimation response could diminish the initial benefits of eCO_2_ in terms of carbon assimilation and growth, particularly under prolonged exposure [82]. Future studies should investigate whether mulberry exhibits similar acclimation responses under long-term eCO_2_ exposure.

### 4.2. eCO_2_ Mitigated the Negative Effects of Drought by Improving Water Use Efficiency

Stomatal responses to eCO_2_ exhibit considerable variability and are influenced by environmental conditions. In this study, lower stomatal conductance and transpiration rates were observed under eCO_2_ compared to aCO_2_ under both well-watered conditions and drought stresses (Figure 2B,C). These findings are consistent with previous studies on various herbaceous and woody species [30,83,84]. The reduction in stomatal conductance under eCO_2_ can be an adaptive mechanism for water conservation while maintaining high levels of photosynthesis, which is associated with decreased stomatal density and an altered stomatal aperture [8]. The Ci/Ca ratio reflects the relationship between stomatal conductance and photosynthetic capacity. Our results showed that Ci/Ca values decreased with declining soil water status under aCO_2_ but remained unchanged under eCO_2_ (Figure 2F), indicating that stomata were sensitive to drought stress under aCO_2_ but exhibited reduced sensitivity or physiological adaptation to eCO_2_. These results indicate that stomatal conductance is not the limiting factor for the increase in the net photosynthetic rate under eCO_2_. Instead, eCO_2_ compensates for the decrease in CO_2_ caused by stomatal closure during drought stress, thereby alleviating the adverse effects of drought stress on plants [85,86]. Plants typically minimize water loss by reducing their leaf transpiration rate as a key adaptive strategy under short-term drought stress [87]. Our results demonstrate that eCO_2_ dramatically improved leaf WUE in mulberry plants subjected to well-watered, moderate-drought, and severe-drought conditions (Figure 2A,C,E). The enhancement in WUE under eCO_2_ can be attributed to the combined effects of reduced stomatal conductance and increased photosynthetic efficiency. Elevated CO_2_ concentrations reduce the stomatal aperture, thereby minimizing water loss via transpiration while ensuring sufficient CO_2_ uptake for photosynthesis. This stomatal regulation is mediated by alterations in guard-cell turgor pressure and the signaling pathways involving abscisic acid and other phytohormones [31]. Furthermore, eCO_2_ enhances the carboxylation efficiency of Rubisco, allowing plants to achieve higher photosynthetic rates with lower stomatal conductance [26]. These physiological adaptations enable mulberry to optimize water use and sustain growth under drought stress, highlighting the critical roles of stomatal behavior and photosynthetic efficiency in improving WUE.

### 4.3. eCO_2_ Mitigated the Negative Effects of Drought by Improving Photosystem II Efficiency

Chlorophyll fluorescence was employed to assess the extent of damage to the photosynthetic apparatus—in particular, to photosystem II (PSII). Under drought stress, impairment of PSII reaction centers impedes primary photochemistry, thereby affecting the process of photosynthetic electron transport. In this study, we observed a reduction in PSII efficiency in mulberry plants due to drought stress, as evidenced by decreased *Φ_PSII_*, ETR, and qP values (Figure 3). Importantly, the detrimental effects of severe drought on *Φ_PSII_*, ETR, and qP were more pronounced under aCO_2_ compared to under eCO_2_ (*Φ_PSII_*, 33% and 25% lower than the control; ETR, 35% and 13% lower; qP, 44% and 4% lower; Figure 3). There was a significant interactive effect between drought stresses and eCO_2_ on *Φ_PSII_*, ETR, and qP (*p* < 0.05, Figure 3), which partially mitigated the damage caused by drought stress. Similar findings have been reported with respect to cucumber [88], coffee plants [30], and wheat [89]. The reduction in PSII efficiency under drought stress directly impacts the photosynthetic electron transport chain, leading to lower energy conversion efficiency and reduced carbon assimilation. This, in turn, limits biomass accumulation and overall productivity in mulberry [90,91]. However, the partial mitigation of PSII efficiency under eCO_2_, as indicated by higher ΦPSII, ETR, and qP values, suggests that mulberry can maintain better photochemical efficiency and energy utilization under combined eCO_2_ and drought conditions. This enhanced photochemical efficiency likely contributes to the observed increase in biomass and productivity under eCO_2_ (Table 2) [41]. Furthermore, a slight decrease in Fv/Fm under eCO_2_+SS indicates that mulberry plants exposed to drought conditions may not have undergone photoinhibition. Therefore, mulberry plants activate non-photochemical quenching and reduced drought-induced PSII efficiency under eCO_2_ (Figure 3). The activation of NPQ under eCO_2_ and drought stress is a protective mechanism that dissipates excess light energy as heat, thereby preventing damage to the photosynthetic apparatus [92]. This mechanism allows mulberry to maintain higher photosynthetic activity and carbon assimilation levels under stress conditions, which are critical for sustaining growth and productivity [93]. The ability of mulberry to optimize energy utilization and protect PSII under combined eCO_2_ and drought stress highlights its potential as a resilient crop for cultivation in water-limited environments [94].

### 4.4. eCO_2_ Mitigated the Negative Effects of Drought by Boosting Mulberry’s Defenses

Drought stress significantly reduces the efficiency of the photosynthetic electron transport chain, leading to a decline in photosynthetic efficiency and an increased electron flow towards molecular oxygen and production of superoxide radicals [95]. The accumulation of ROS is detrimental to biomolecules such as nucleic acids, proteins, chloroplast pigments, etc. [96,97]. In response to drought-induced oxidative stress, plants develop a complex antioxidant system to regulate cellular redox status. CAT, POD, and SOD serve as the first line of defense against reactive oxygen species. In this study, the activities of CAT, POD, and SOD were significantly increased in both leaves and roots under drought stress, which is consistent with previous results [10,98]. Furthermore, eCO_2_ further improved the activities of CAT, POD, and SOD in leaves and/or roots under drought stress conditions (Figure 5), in line with other studies [12,76]. The impact was more pronounced for SOD under moderate drought and for POD under severe drought, indicating distinct responses in antioxidant metabolism. The mitigation of ROS formation under eCO_2_ can be attributed to increased electron consumption in C fixation, reducing the diversion of electrons towards ROS-generating pathways such as photorespiration or the Mehler reaction [99,100]. Enhanced carbon assimilation under eCO_2_ provides more resources for the synthesis of antioxidant enzymes and osmoprotectants, thereby mitigating oxidative damage and supporting cellular functions under drought stress. Soluble sugars, acting as osmolyte and metabolite signaling molecules, play a crucial role in modulating plant responses to stress [82]. The elevated concentration of soluble sugars under eCO_2_ observed in this study (Figure 4C,D) indicates enhanced C assimilation, which improves turgor pressure through osmotic adjustment. This finding is consistent with a previous study showing that soluble sugars (such as glucose and sugar alcohols) contributed to increased root biomass in *Sorghum bicolor* and alleviated drought stress under eCO_2_ [101]. The accumulation of soluble sugars under eCO_2_ not only supports osmotic adjustment but also provides energy for growth and stress responses [79]. Proline serves as both a stress marker and an osmoprotectant, implying that plants may accumulate more proline under drought stress or less proline when experiencing reduced stress levels [102]. Our data demonstrate that eCO_2_ decreased proline content in both leaves and roots under drought stress (Figure 4A,B). Hence, the reduced accumulation of proline in plants exposed to eCO_2_ suggests that excessive proline buildup is not necessary to withstand drought stress [103]. The reduced proline accumulation under eCO_2_ indicates that mulberry experiences less severe drought stress due to improved water use efficiency and antioxidant defense. This allows the plant to allocate more resources towards growth and productivity rather than stress mitigation [104]. These changes in antioxidant metabolites and osmolytes clearly indicate that mulberry plants modify their biochemical system to counteract the detrimental effects of drought stress under eCO_2_.

### 4.5. eCO_2_ Mitigated the Negative Effects of Drought by Regulating NPK Partitioning

Drought stress can inhibit plant growth by reducing nutrient uptake and affecting the relocation of N, P, and K in plants [105,106]. A meta-analysis revealed that drought stress results in decreased levels of N and P in plant tissues, as well as reduced nutrient absorption from soil [39]. In this study, the concentrations of N, P, and K in stems and roots were significantly lower under drought stress compared to well-watered conditions under aCO_2_, and a greater decrease was observed under moderate drought than under severe drought (Table 2). The impacts of drought on N, P, and K concentrations were more pronounced in roots and stems compared to leaves, indicating that soil drought negatively affected the nutrient uptake capacity of roots and stems. The decline in nutrient levels could be attributed to decreased photosynthesis and transpiration rates under drought conditions, resulting in reduced nutrient uptake (Figure 2 and Figure 8). This coincided with an increase in the allocation of N, P, and K to leaves and a decrease in their allocations to stems and/or roots (Figure 7). However, these findings are inconsistent with studies suggesting drought may accelerate N transfer from green leaves to sink tissues such as roots or grains [107]. The increased allocation of N, P, and K to leaves observed in this study could be a mechanism of drought avoidance through the synthesis and accumulation of osmoprotectants and antioxidant enzymes, which are predominantly found in higher concentrations in leaves than in roots (Figure 5 and Figure 6), as further supported by a significantly positive correlation between plant N, P, and K concentrations and antioxidant enzyme activities (Figure 9).

Fortunately, the detrimental impacts of drought on the concentrations of N, P, and K in stems and roots were mitigated under eCO_2_ compared to under aCO_2_, and even drought stress increased root P concentrations under eCO_2_ (Table 1). Previous studies have reported a decline in plant-tissue N due to CO_2_ enrichment [46,61,62,108], which subsequently affects other components involved in N metabolism, such as proline levels [109]. Similar results were observed in this study (Table 1 and Figure 4B,E). Under both well-watered and severe-drought conditions, eCO_2_ led to decreases in N and P contents in mulberry tissues, but the reduction was less pronounced under severe drought than under well-watered conditions (Table 1). This phenomenon may be attributed to the nutrient dilution effect, where increased biomass production under eCO_2_ leads to lower concentrations of essential nutrients such as N and P in plant tissues [110]. This nutrient dilution could have implications for the nutritional quality of mulberry leaves, which are critical for silkworm feeding and sericulture productivity. Future research should explore strategies to mitigate nutrient dilution effects, such as optimized fertilization practices or genetic improvements in nutrient use efficiency.

Furthermore, eCO_2_ induced allocations of N, P, and K to root tissues under drought stresses (Figure 8). This adaptation to eCO_2_ facilitates greater allocation to the expanded root system rather than the photosynthetic apparatus of green tissues, optimizing energy costs [111]. Thus, eCO_2_ and drought stress may increase nutrient use efficiency by regulating nutrient reallocation [112].

### 4.6. eCO_2_ Mitigated the Negative Effects of Drought by Improving Nutrient Uptake and Stimulating Soil Enzyme Activities

Most studies on eCO_2_ and/or drought have primarily focused on plant characteristics, with limited research conducted on the feedback between plant nutrient uptake and soil nutrient supply. It is widely acknowledged that soil nutrient reserves play a crucial role in determining plant growth. However, the impacts of eCO_2_ and/or drought on the soil nutrient pool and plant requirements remain unpredictable. Our results demonstrate that plants exhibited reduced uptake of N, P, and K when subjected to decreased soil moisture (Figure 6), resulting in higher contents of available forms of these nutrients in soil (Figure 8). Reduced nutrient uptake during drought may be attributed to factors such as diminished mineralization-mediated nutrient supply, as well as reduced nutrient diffusion and mass flow in soil [113]. Additionally, altered dynamics of root nutrient uptake induced by drought can also contribute to decreased acquisition of essential elements by plants [114]. In contrast, our results indicate that eCO_2_ significantly enhanced N, P, and K accumulation in mulberry plants under both well-watered and drought-stressed conditions (Figure 6), leading to decreased levels of AN, AP, and AK in soil, except for AN under drought stress (Figure 8). These findings suggest that eCO_2_ ameliorated the adverse impacts of drought by improving nutrient uptake. Consistent with our findings, previous studies have displayed similar effects; for example, 700 ppm eCO_2_ upregulated gene expression related to nutrient uptake proteins, enhancing the rates of N and P assimilation [110].

The activities of soil enzymes indicate microbial functions in decomposing organic matter and mobilizing nutrients [48]. In this study, we observed significant decreases in sucrase, urease, and phosphatase activities under drought conditions (Figure 8D–F), consistent with previous studies that have reported negative correlations between soil moisture and enzymatic activities [45,51,115]. The reduction in enzyme activities could also be attributed to the low microbial biomass and physiology [116] or decreased enzyme production and turnover rates [117]. Drought-induced inhibition of plant growth and reductions in rhizodeposition result in decreased substrate availability. However, our study revealed significantly higher activities of soil sucrase, urease, and phosphatase under eCO_2_ than under aCO_2_ as soil water content declined (Figure 8D–F), indicating a positive correlation between eCO_2_ levels and soil enzyme activities (Figure 9). Similar increases in enzyme activities have been reported in herbaceous and woody soils under 550–700 ppm eCO_2_ [47,49,118]. A two-year FACE study conducted in a semi-arid grassland at the United States Department of Agriculture’s Agricultural Research Service High Plains Grasslands Research Station demonstrated that 600 ppm eCO_2_ had a positive effect on soil C, N, and P cycling by increasing the abundance of microbial functional genes, which were closely correlated with the increase in soil moisture [119]. The increase in soil enzyme activities is directly related to the plant root exudates [120]. Therefore, eCO_2_ could accelerate C turnover in the rhizosphere and stimulated soil enzyme activities, thereby influencing the microbial biomass and microbial activity that was not tested in this study, which might ultimately have potential to ameliorate the adverse impacts of drought.

However, the long-term sustainability of eCO_2_ effects on mulberry growth and productivity remains uncertain. While eCO_2_ can enhance nutrient uptake and soil enzyme activities in the short term, prolonged exposure to elevated CO_2_ may alter soil nutrient dynamics and microbial activity, potentially affecting nutrient availability and plant health over time [121]. For example, increased carbon inputs under eCO_2_ may accelerate soil organic matter decomposition, leading to changes in nutrient cycling and microbial community composition [122]. Future studies should investigate the long-term impacts of eCO_2_ on mulberry growth, nutrient cycling, and ecosystem interactions to fully understand its potential benefits and limitations.

## 5. Conclusions

**Figure 10:**
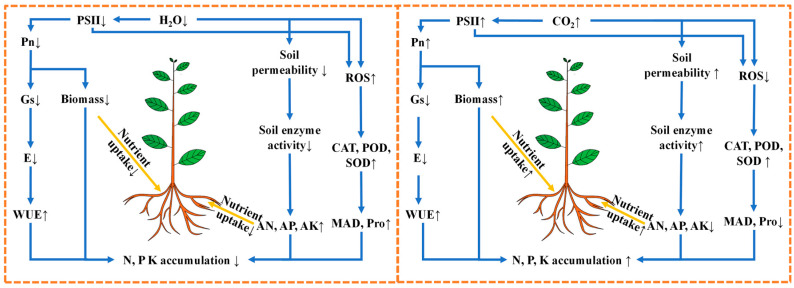
Comparative schematic model showing how mulberry plants can respond to drought and elevated CO_2_ concentrations. PSII, photosystem-II; Pn, net photosynthetic rate; gs, stomatal conductance; E, transpiration rate; WUE, water use efficiency in leaves; N, nitrogen; P, phosphorus; K, potassium; AN, available nitrogen; AP, available phosphorus; AK, available potassium; ROS, reactive oxygen species; CAT, catalase; POD, peroxidase; SOD, superoxide dismutase; MDA, malondialdehyde; Pro, proline. An upward arrow represented an increase, whereas a downward arrow signified a reduction.

We conducted a comprehensive study on the responses of mulberry plants to drought under eCO_2_. As shown in Figure 10, eCO_2_ promotes mulberry’s photosynthesis and WUE under drought. It also mitigates the adverse effects of drought on ETR and ΦPSII, thereby decreasing damage to photosystems and ROS accumulation in mulberry under drought stress. Moreover, eCO_2_ enhanced the tolerance of mulberry to drought stress by increasing activities of antioxidant enzymes such as SOD, POD, and CAT, and the contents of osmolytes such as proline, malondialdehyde, and soluble sugars in both leaves and roots. Furthermore, eCO_2_ stimulated the activities soil enzymes under such as soil sucrase, urease, and phosphatase, under drought, improving soil fertility. This stimulation facilitated nutrient uptake of soil N, P, and K, leading to their increased accumulation in mulberry tissues while promoting their allocation to an expanded root system under drought. The enhanced photosynthetic capacity, reduced oxidative stress, and increased nutrient absorption induced by eCO_2_ can effectively mitigate the detrimental impact of drought on mulberry growth by augmenting both above- and below-ground biomass production. Our findings provide crucial insights to guide water management strategies in mulberry plantations while increasing leaf growth and productivity through the regulation of N, P, and K nutrition under future conditions of elevated CO_2_ and drought.

## Figures and Tables

**Table 1:** Variations in tissue nitrogen (N), phosphorus (P), and potassium (K) concentrations of mulberry under ambient (aCO_2_) and elevated CO_2_ (eCO_2_) under well-watered, moderate-drought, and severe-drought conditions.

Variable	*p*-Value			CO_2_ Treatment	Water Treatment			Moderate Water Deficit Effect (%)	Severe Water Deficit Effect (%)
	CO_2_	Water	C*W		Well-Watered	Moderate Drought	Severe Drought		
Leaf N concentration (mg g^−1^)	0.001	0.055	0.276	aCO_2_	34.5 ± 0.6 y,a	35.0 ± 1.1 y,a	38.0 ± 0.1 x,a	+1.3	+10.1
				eCO_2_	32.4 ± 0.7 x,b	31.4 ± 0.6 x,b	33.0 ± 1.4 x,b	−3.2	+1.8
				CO_2_ effect (%)	−6.1	−10.1	−13.0		
Stem N concentration (mg g^−1^)	0.042	0.001	0.159	aCO_2_	16.2 ± 0.4 x,a	13.3 ± 0.5 y,a	13.4 ± 0.8 y,a	−17.9	−17.2
				eCO_2_	14.5 ± 0.4 x,b	11.3 ± 0.5 y,b	13.11 ± 0.8 x,a	−21.7	−9.6
				CO_2_ effect (%)	−10.5	−15.9	−2.2		
Root N concentration (mg g^−1^)	0.308	0.526	0.045	aCO_2_	16.5 ± 0.6 x,a	14.0 ± 0.9 y,a	14.6 ± 0.3 y,a	−15.6	−11.2
				eCO_2_	13.9 ± 0.5 x,b	15.0 ± 0.9 x,a	14.5 ± 0.3 x,a	+8.3	+4.8
				CO_2_ effect (%)	−15.9	+7.6	−0.9		
Leaf P concentration (mg g^−1^)	0.007	0.136	0.283	aCO_2_	1.30 ± 0.06 x,a	1.18 ± 0.04 y,a	1.28 ± 0.04 x,a	−9.1	−0.9
				eCO_2_	1.08 ± 0.08 y,b	1.10 ± 0.03 y,b	1.20 ± 0.03 x,b	+3.2	+8.2
				CO_2_ effect (%)	−16.7	−6.6	−5.8		
Stem P concentration (mg g^−1^)	0.012	0.010	0.226	aCO_2_	0.99 ± 0.04 x,a	0.84 ± 0.02 y,a	0.86 ± 0.04 y,a	−15.1	−13.1
				eCO_2_	0.85 ± 0.04 x,b	0.72 ± 0.04 y,b	0.85 ± 0.06 x,a	−14.7	−0.8
				CO_2_ effect (%)	−14.5	−14.2	−0.9		
Root P concentration (mg g^−1^)	0.044	0.039	0.005	aCO_2_	0.95 ± 0.04 x,a	0.74 ± 0.05 y,a	0.95 ± 0.06 x,a	−22.0	−0.4
				eCO_2_	0.70 ± 0.03 y,b	0.84 ± 0.06 x,a	0.87 ± 0.02 x,b	+20.8	+26.1
				CO_2_ effect (%)	−26.6	+12.6	−8.4		
Leaf K concentration (mg g^−1^)	0.294	0.003	0.271	aCO_2_	20.0 ± 0.4 y,a	19.2 ± 0.8 y,a	21.6 ± 0.3 x,a	−3.7	+8.4
				eCO_2_	18.8 ± 0.3 z,b	19.7 ± 0.3 y,a	21.15 ± 0. 6 x,a	+4.8	+10.2
				CO_2_ effect (%)	−6.1	+2.7	−2.7		
Stem K concentration (mg g^−1^)	0.677	0.106	0.785	aCO_2_	7.6 ± 0.6 x,a	6.0 ± 0.2 y,a	7.2 ± 0.7 x,a	−21.1	−5.6
				eCO_2_	7.2 ± 0.8 x,a	6.3 ± 0.1 x,a	6.8 ± 0.6 x,a	−12.7	−6.0
				CO_2_ effect (%)	−5.3	+4.5	−5.8		
Root K concentration (mg g^−1^)	0.802	0.150	0.108	aCO_2_	8.3 ± 0.6 x,a	6.5 ± 0.7 y,a	8.2 ± 0.1 x,a	−22.2	−1.1
				eCO_2_	7.5 ± 0.4 x,a	7.3 ± 0.5 x,a	7.6 ± 0.5 x,a	−2.0	+1.8
				CO_2_ effect (%)	−10.0	13.1	−7.2		

Data (means ± SE, n = 6) followed by different letters indicate significant differences between CO_2_ treatments for the same water regime (a, b) and among water treatments for the same CO_2_ concentration (x, y, z) at *p* < 0.05. Statistical comparisons (two-way ANOVA) between water regimes and CO_2_ treatments, as well as their interaction (water × CO_2_), are presented for each variable.

**Table 2:** Variations in plant tissue biomass production of mulberry plants under ambient CO_2_ (aCO_2_); elevated CO_2_ (eCO_2_); and well-watered, moderate-drought, and severe-drought conditions.

Variable	*p*-Value			CO_2_ Treatment	Water Treatment			Moderate Water Deficit Effect (%)	Severe Water Deficit Effect (%)
	CO_2_	Water	C*W		Well-Watered	Moderate Drought	Severe Drought		
Leaf biomass (g plant^−1^)	0.002	0.000	0.981	ACO_2_	37.5 ± 1.8 x,b	32.9 ± 1.0 y,b	28.6 ± 2.9 z,b	−12.4	−23.8
				ECO_2_	47.3 ± 0.4 x,a	43.4 ± 0.5 y,a	38.8 ± 2.7 z,a	−8.3	−18.0
				CO_2_ effect (%)	+26.1	+32.0	+35.7		
Stem biomass (g plant^−1^)	0.000	0.000	0.037	ACO_2_	20.3 ± 0.9 x,b	16.5 ± 0.9 y,b	10.0 ± 0.6 z,b	−18.9	−50.6
				ECO_2_	24.4 ± 1.4 x,a	21.3 ± 0.8 y,a	18.4 ± 0.9 z,a	−12.6	−24.4
				CO_2_ effect (%)	+20.1	+29.4	+83.7		
Root biomass (g plant^−1^)	0.000	0.001	0.574	ACO_2_	19.8 ± 0.9 x,b	16.9 ± 0.8 y,b	14.3 ± 1.2 z,b	−14.8	−27.8
				ECO_2_	23.9 ± 0.4 x,a	20.1 ± 0.8 y,a	16.5 ± 0.8 z,a	−16.0	−30.9
				CO_2_ effect (%)	+20.4	+18.8	+15.3		
Total biomass (g plant^−1^)	0.000	0.000	0.876	ACO_2_	77.7 ± 3.4 x,b	66.2 ± 1.3 y,b	53.0 ± 3.8 z,b	−14.7	−31.8
				ECO_2_	95.6 ± 2.1 x,a	84.8 ± 1.1 y,a	73.8 ± 14.2 z,a	−11.3	−22.8
				CO_2_ effect (%)	+23.1	+28.0	+39.2		
Root : shoot ratio	0.027	0.164	0.080	ACO_2_	0.34 ± 0.01 x,a	0.34 ± 0.01 x,a	0.37 ± 0.04 x,a	−0.3	+9.4
				ECO_2_	0.33 ± 0.01 x,a	0.31 ± 0.02 x,b	0.28 ± 0.02 x,b	−6.8	−13.1
				CO_2_ effect (%)	−2.9	−9.2	−22.9		

Data (means ± SE, *n* = 6) followed by different letters indicate significant differences between CO_2_ treatments for the same water regime (a, b) and among water treatments for the same CO_2_ concentration (x, y, z) at *p* < 0.05. Statistical comparisons (two-way ANOVA) between water regimes and CO_2_ treatments, as well as their interaction (water × CO_2_), are presented for each variable.

## Data Availability

Data is contained within the article.

## Abbreviations

The following abbreviations are used in this manuscript:

aCO_2_	Ambient CO_2_
eCO_2_	Elevated CO_2_
Ci	Intercellular CO_2_ concentration
N	Nitrogen
P	Phosphorus
K	Potassium
WW	Well-watered
MS	Moderate drought stress
SS	Severe drought stress
WUE	Water use efficiency
ROS	Reactive oxygen species
CAT	Catalase
POD	Peroxidase
SOD	Superoxide dismutase
MAD	Malondialdehyde
TAB	Thiobarbituric acid
NBT	Nitrogen blue tetrazolium
EDTA-Na_2_	Ethylenediaminetetraacetic acid disodium salt
KMnO_4_	Potassium permanganate
SOC	Soil organic carbon
AN	Soil available nitrogen
AP	Soil available phosphorus
AK	Soil available potassium
F0	Initial fluorescence yield under the dark-adapted stage
Fm	Maximum fluorescence yield under the dark-adapted stage
F0′	Minimum fluorescence yield under the light-adapted stage
Fm′	Maximum fluorescence under the light-adapted stage
ΦPSII	Effective quantum yield of PSII photochemistry
Fv/Fm	Maximal quantum yield of PSII photochemistry
Fv′/Fm′	Actual photosynthetic efficiency of PSII under illumination
qP	Photochemical quenching coefficient
NPQ	Non-photochemical quenching coefficient
ETR	PSII electron transport rate
SEM	Structural equation modeling
